# American Veterinary College

**Published:** 1882-04

**Authors:** 


### AMERICAN VETERINARY COLLEGE.

---

The annual exercises of the American Veterinary College were held on the evening of March 6. The following is a list of graduates: Gabriel Smith Agesborg, Vermilion, Dakota; Joseph Ferdinand Autenreith, Jersey City; William Stoughton Devoe, New York City; George Sherbrooke Houghton, New York City; Lester Heard Howard, Clinton, Mass.; James Samuel Kemp, Jr., Holbrook, L. I.; William Manz, New York City; William Howard Martenet, Baltimore, Md.; Charles Leroy Moulton, Manchester, N. H.; Frank Eugene Risley, Waterville, N. Y.; Horace Ward Atwood, B.S., North Orange, Mass.; August Joseph Jeannin, Navarre, Ohio; George Henry Keefer, M.D., Hillsdale, Mich.; John Albert Leighton, New York

City; Everett Woodhull Rowland, Miller's Place, N. Y.; Ward Beecher Rowland, Media, Pa.; Fred. Saunders, Salem, Mass.; Frank Traver, Rhinebeck, N. Y. The medals awarded are as follows: Best general examination, Board of Trustees prize, gold medal, Joseph S. Kemp, Jr.; second best; examination, Alumni prize, standard veterinary works, Lester H. Howard; best practical examination, New York State Veterinary Society prize, gold medal, Joseph S. Kemp, Jr.; silver medal, given by Dr. Liautard, for best general examination in junior class on Anatomy, given to W. B. C. Noyes.

